# Supplementary material for: A qualitative study to explore healthcare providers’ perspectives on barriers and enablers to early detection of breast and cervical cancers among women attending primary healthcare clinics in Johannesburg, South Africa
Source: PLOS Glob Public Health. 2023 May 9;3(5):e0001826. doi: 10.1371/journal.pgph.0001826 (PMC10168575; doi:10.1371/journal.pgph.0001826)
Supplement: S1 Table — (DOCX) [file pgph.0001826.s001.docx]

**S1 Table: Coding Tree: Highlights the coding process and data analyses process**

| THEME | SUB-THEMES | CODES | **DESCRIPTIONS** |
| --- | --- | --- | --- |
| **ACTIVITIES IN PLACE FOR EARLY CANCER DETECTION** |  |  | To identify screening procedures conducted at primary healthcare for the detection of cancers |
|  | Breast |  |  |
|  | Cervical |  |  |
| **AWARENESS AND KNOWLEDGE ABOUT COMMON CANCER EARLY DETECTION GUIDELINE** |  |  | To understand the level of awareness and knowledge of common cancers and early detection guidelines |
|  | Awareness of NDoH guidelines |  |  |
|  | Detection and management of common cancers at PHC | Breast |  |
|  |  | Cervical |  |
|  |  |  |  |
|  | Drs and nurses’ level of knowledge about cancer |  |  |
|  | Opinions about routine screening for early cancer symptoms |  | To explore participants opinions about carrying out cancer screening procures routinely |
|  |  | Description of clinical breast examination process |  |
|  |  | Description of cervical screening procedure |  |
|  | Perceptions on standard of care provided to patients |  |  |
|  | Perceptions on standard of care provided to patients |  |  |
|  | Referral pathway |  | To gain an insight on the common cancer’s referral pathways |
| **BARRIERS TO ROUTINE BREAST AND CERVICAL CANCER SCREENING** | Healthcare provider barriers | Lack of knowledge of cancer and early detection guidelines |  |
|  |  | No private rooms-facilities or equipment’s to examine patents |  |
|  |  | Not enough provider competency for pap smear procedure |  |
|  |  | Not enough providers for high patient volume |  |
|  |  | Patients hopping from one clinic to another |  |
|  |  | Rely on patient to report with symptoms |  |
|  |  | reporting system |  |
|  |  | staff attitude |  |
|  |  | Staff attitude towards learning |  |
|  |  | Staff being rude to patients attributed to burnout |  |
|  |  | Staff rotation - difficulty for healthcare provider to develop skill competence |  |
|  | Patient barriers | Cultural beliefs |  |
|  |  | fear of cancer diagnosis |  |
|  |  | Lack of cancer knowledge |  |
|  |  | Myths and misconceptions about cancer |  |
|  |  | Patient attitude |  |
|  |  | Patients fear of the pap smear procedure being painful |  |
|  |  | Patients presenting late at the clinic |  |
|  |  | Patient’s social factors |  |
|  |  | Patients view healthcare services as curative and not preventative |  |
|  |  | Uncomfortable to be attended by someone they know |  |
|  |  | Use home remedies or alternative treatment first |  |
| **FACILITATORS TO BREAST AND CERVICAL SCREENING** | Community education on early signs and symptoms |  | To explore barriers to breast and cervical cancer early detection |
|  | In service training on policies |  |  |
|  | Increase nursing staff and provide them with checklist |  |  |
|  | Next visit documented on appointment card |  |  |
|  | Provision of fully equipped screening rooms |  |  |
|  | Policies in place |  |  |
|  | Symptoms probing and patient education |  |  |
| **MOTIVATION REQUIRED TO GET BUY IN FROM CLINIC STAFF** |  |  | To understand what would be required to motivate primary healthcare workers to perform cancer screening routinely to patients attending the primary healthcare |
| **MOTIVATORS REQUIRED TO BE CHAMPION FOR GUIDELINES** |  |  |  |
| **PERCEPTION OF OWN CANCER KNOWLEDGE** |  |  | Participants own assessment on their knowledge about common cancer |
| **RECOMMENDATIONS** |  |  | Suggestions on what can be done to educate primary healthcare worker on early cancer guidelines and implement routine cancer screening |
| **REQUIRED RESOURCES FROM DISTRICT OR NATIONAL** |  |  | To explore resources required by the primary healthcare worker to implement early cancer detection guideline |
| **STAFF ROTATION** | Advantages |  | To gain an understanding on how staff is rotated and impacted on early cancer screening |
|  | Disadvantaged |  |  |
|  |  |  |  |
