# Supplementary material for: A qualitative study to explore healthcare providers’ perspectives on barriers and enablers to early detection of breast and cervical cancers among women attending primary healthcare clinics in Johannesburg, South Africa
Source: PLOS Glob Public Health. 2023 May 9;3(5):e0001826. doi: 10.1371/journal.pgph.0001826 (PMC10168575; doi:10.1371/journal.pgph.0001826)
Supplement: Attachment — Submitted filename: RebuttalLetter.pdf [file pgph.0001826.s004.pdf]

12th Floor, New Nurses Home
Chris Hani Baragwanath Academic Hospital
Diepkloof
1864
+27 11 989 9831

Rebuttal letter for manuscript: PGPH-D-22-01548
Title: A qualitative study to explore healthcare providers' perspectives on barriers and enablers to early detection of breast and cervical cancers among women attending primary healthcare clinics in Johannesburg, South Africa

Dear Professor Moodley

Thank you very much for the manuscript review and for affording us the opportunity to respond to journal and reviewer requirements and queries, which we do so below.

Editorial comment

In addition for this qualitative study, please complete the COREQ checklist.

Response

The COREQ checklist has been completed and uploaded.

Reviewer 1

Reviewer #1: On the whole the reviewers' comments have been addressed. However the results section is still a little misleading as the headings do not reflect that these are healthcare providers views (rather than patients). Suggest these are altered e.g. "Healthcare providers think patients lack cancer related knowledge"; "Healthcare provides think patients do not recognise cancer signs and symptoms"; "Healthcare providers think patients perceive cancer as being associated with white people"; "Healthcare providers believe patients have misconceptions about cervical cancer screening procedures"; "Healthcare providers think patients view healthcare services as curative not preventative".

Response

All headings have been revised as suggested above.

Response

Relevant heading have been changed to reflect the healthcare providers views and not patient views, the following changes have been made:

1. **Line 430:** "Patients lack cancer-related knowledge" has been edited and now reads as <u>"Healthcare providers think patients lack cancer-related knowledge</u>"

2. **Line 431:** "Patients do not recognize cancer signs and symptoms" has been edited and now reads as "<u>Healthcare providers think that patients do not recognize cancer signs and symptoms</u>"

3. **Line 458-459:** "Patients perceive cancer as being associated with white people" has been edited and now reads as "Healthcare providers think patients perceive cancer as being associated with white people"

4. **Line 470-471:** "Patients have misconceptions about cervical cancer screening procedure' has been edited and now reads as "Healthcare providers believe patients have misconceptions about cervical cancer screening procedure"

5. **Line 509-510:** "Healthcare providers think patients view healthcare services as curative and not preventative" has been edited and now reads as "Healthcare providers think patients view healthcare services as curative and not preventative"

Reviewer comment
This caution should be applied in the discussion too, particularly lines 572 – 575.

Response

Line 580-583 (initially was line 572-572) in the discussion section has revised as well to reflect views made by healthcare providers. "Providers reported that patients to had poor cancer risk and symptoms knowledge exacerbated by perceptions of low cancer risk and screening procedures perceived as being painful. Women prioritised self-medication and traditional healing, utilising health services only when they felt very ill." has been revised and now reads as follows: "Providers reported that they perceive patients to have poor cancer risk and symptoms knowledge exacerbated by perceptions of low cancer risk and screening procedures perceived as being painful. Providers believe that women prioritised self-medication and traditional healing, utilising health services only when they felt very ill."

Kind regards

Janan Dietrich, PhD, Psychology (she/her)
Director: Bio-Behavioural Research: **PERINATAL HIV RESEARCH UNIT**
Associate Professor: Clinical Medicine Department, Faculty of Health Sciences, University of the Witwatersrand
Executive Director: African Social Sciences Unit of Research and Evaluation (ASSURE)
Early Career Investigator: South African Medical Research Council
E-mail: dietrichj@phru.co.za
Peer reviewed publications: Google scholar profile
Office Landline: 011 989 9831 (Lerato – Research Associate)

African Social Sciences Unit of Research and Evaluation (ASSURE) is a research unit and operates as a division of the Wits Health Consortium

The Wits Health Consortium (Pty) Limited (WHC) is wholly-owned by the University of the Witwatersrand.
(Pty) Ltd. Company Registration No: 97/15443/07

Executive Directors: Prof Janan Dietrich and Dr Nellie Myburgh
