# Supplementary material for: A qualitative study to explore healthcare providers’ perspectives on barriers and enablers to early detection of breast and cervical cancers among women attending primary healthcare clinics in Johannesburg, South Africa
Source: PLOS Glob Public Health. 2023 May 9;3(5):e0001826. doi: 10.1371/journal.pgph.0001826 (PMC10168575; doi:10.1371/journal.pgph.0001826)
Supplement: Attachment — Submitted filename: Responsetoreviewers.docx [file pgph.0001826.s003.docx]

**Rebuttal letter for manuscript**: PGPH-D-22-01548

**Title**: A qualitative study to explore healthcare providers’ perspectives on barriers and enablers to early detection of breast and cervical cancers among women attending primary healthcare clinics in Johannesburg, South Africa

Dear Professor Moodley

Thank you very much for the manuscript review and for affording us the opportunity to respond to journal and reviewer requirements and queries, which we do so below. All changes have been track changed. As is required we submitted two versions of the manuscript: Revised version with track changes and a clean version.

Kind regards

Janan Dietrich, PhD, Psychology (she/her)

**Director:** [Bio-Behavioural Research](https://www.phru.co.za/about-us/12-our-research/researchers/98-janan-dietrich): **PERINATAL HIV RESEARCH UNIT**

**Associate Professor:** Clinical Medicine Department, Faculty of Health Sciences, University of the Witwatersrand

**Executive Director**: [African Social Sciences Unit of Research and Evaluation (ASSURE)](http://assureafrica.com/)

**E-mail:** [dietrichj@phru.co.za](mailto:dietrichj@phru.co.za)

**Office Landline:** 011 989 9831 (Lerato – Research Associate)

**Journal Requirements**

1. Please amend your detailed Financial Disclosure statement. This is published with the article. It must therefore be completed in full sentences and contain the exact wording you wish to be published.

Our financial disclosure statement is as follows: The authors declare that they have no competing interests.

a. State what role the funders took in the study. If the funders had no role in your study, please state: “The funders had no role in study design, data collection and analysis, decision to publish, or preparation of the manuscript.”
b. If any authors received a salary from any of your funders, please state which authors and which funders.

The funders had no role in study design, data collection and analysis, decision to publish, or preparation of the manuscript. Gugulethu Tshabalala and Janan Dietrich received partial salary funding from the funder Bristol Myers Squibb Foundation-Multi-Sector Coalition in Implementation Research in Global Oncology (CIRGO) for conducting the study.

**Any reference to line numbers uses the clean version of the manuscript.**

Additional Editor Comments (if provided):

In addition to addressing the Reviewers comments, could the authors please also address the following:

1. Line 90 “ bare” should be “bear”

Line 90 “bare” has been corrected to “bear” (now line 91) in both marked and unmarked manuscript revisions.

2. The authors refer to Black and White patients throughout the study. Use of such terms can lead to unintentional bias. Could the authors please justify use of this terminology, making clear the origins of these terms and that these racial classifications are social constructs without biological meaning.

We agree with the editors comment and believe it is necessary to provide an explanation. We have done the following:

i) Omitted the word “Black” from line 169 in the Introduction.
ii) The term Black is now used only within the context of participants’ responses within the results and discussion sections.
iii) We have included the following statement on lines 264-266: In the context of this qualitative study’s results, participants refer to Black and White groups. These classifications are not racial, but rather social constructs of South African historical Apartheid origin, without biological meaning.

3. The use of the term “pap” throughout the article needs to be revised. The correct abbreviation is “Pap” Could the authors please use the full term the first time it is used.

We thank the editor for pointing out this error. We have revised line 171 to include the words “**Papanicolaou test (Pap test)”**. Thereafter, throughout the manuscript “pap” was replaced by “Pap”

4. In the Western Cape study referred to in Line 606 providers did consider cancer as a differential diagnosis but highlighted the difficulty of distinguishing between symptoms of infectious diseases and cervical cancer at a primary care level which is important to consider given the high burden of STIs in the setting. It pointed to a need to support primary care providers in assessing symptomatic patients.

We thank the editor for this correction. We have replaced the text as follows – lines 594-597: “In a recent study in the Western Cape of SA, Moodley et al^28^ highlighted the difficulty for primary care providers to distinguish symptoms of infectious diseases from those of cervical cancer in settings of high infectious disease prevalence. They pointed to a need to support primary care health care providers in assessing symptomatic patients.”

**Additional author revisions**

We revised our affiliations, our funding statement, as well as the results and conclusion components of the abstract.

Reviewer #1

This is an important study with a clear rationale and detailed account of methods. The study provides useful, insightful and sometimes concerning insights into barriers to early cancer detection. The authors have embedded findings in existing model of behaviour and this works well.

There are a few issues that would benefit from consideration:

**Reviewer**
Make sure the text is clear that the findings refer to the providers’ views of patients/women (e.g. lines 587, 624, 619). Women may not necessarily hold these views/behave this way.
Similarly, suggest headings in Figure 2 are ‘Providers perspectives of policy’, ‘Providers perspectives of organisation and staff’ ‘Providers perspective of individual and socio-cultural issues’

**Response**

We thank reviewer # 1 for this instruction. We have added lines 518-519 which states “These results refer to the providers’ views of patients and/or women and may not necessarily reflect the views nor behaviours of patients/women ”.

Additionally, we added lines 648-650 to the limitations as follows: “The results of this study present provider experiences and perceptions of patients and/or women. Patients/women may not necessarily hold these views or behave in the way referred to by providers.”

Figure 2 has been revised to with headings amended to state provider perspectives.

**Reviewer**
The section on ‘enablers for early breast and cervical cancer detection’ felt quite repetitive as it highlighted opposites of the previously described barriers (e.g. barrier = lack of training; enabler = training opportunities). Suggest this section is merged with the previous section.

We thank the reviewer for this suggestion and have duly shortened and integrated the enablers section into the barriers section, with track-changes.

Figure 2 is useful but would benefit from better formatting to align boxes etc

We have aligned boxes in figure 2 and emphasized the provider perceptions and views with adjusted headings.

The reference section needs attention. Check the references are numbered in the order they appear in the text and numbers correspond to the correct reference in the list. Also check all are in the list: references 20-23 and 36-38 appear missing from the list.

The Reference section has been corrected and double-checked. There are now 30 references in the revised manuscript.

Other minor points:

Line 547 ‘burnout’ not ‘burn out’

We have corrected this error (now line 534).

Spell out COM-B acronym and give brief overview of this model

We have inserted lines 603-607: “Utilising the socioecological^29^ and capability, opportunity and motivation model of behaviour (COM-B) models, we firstly grouped our findings and explored pathways that influence low screening provision^30^. The COM-B model identifies what needs to change for a behavioural intervention to be effective. The COM-B model identifies three factors that need to be present for any behaviour to occur: capability, opportunity and motivation.”

Check if LMIC refers to low-to-middle income countries or low and middle income countries.

In the context of this study, LMIC refers to Low- and -Middle- Income- Countries and we have made the change accordingly (lines 89, 146).

Reviewer #2: Thank you for the opportunity to review this manuscript, which uses data from qualitative interviews with health providers and clinic managers in Soweto, South Africa to report on factors at multiple levels – policy, organisational, and individual (providers and users) – that may explain low breast and cervical cancer screening volumes at primary care clinics in Soweto, South Africa, and possible solutions for addressing the identified barriers. I commend the authors for addressing these relevant questions because despite enabling policies, breast examination and cervical screening and pre-cancer treatment coverage remain low in South Africa, and solutions are needed. The manuscript has some strengths and potential to contribute to the literature. It also has weaknesses that need addressing before it is fit to publish. Below are suggestions for improving the quality of the manuscript.

As an overall comment, it is problematic that the manuscript draws conclusions about patient-side barriers based on provider perceptions. This approach does not adequately represent user / patient voices in the health system. The manuscript also draws some conclusions that are not supported by the data and seem to be over-reach. The manuscript does however report some useful findings, but its main message must be articulated better and coherence between the problem, aim and results must be improved if it is to appeal to a broader South African and international audience.

We thank the reviewer for this comment. We have clarified that the views and perceptions are those of the providers within the revised manuscript as follows:-

i) Results and conclusion sections of the abstract emphasise that findings are from a provider perspective and need to be confirmed among other stakeholders
ii) We inserted the line 518: “These results refer to the healthcare providers’ views of patients and/or women and do not necessarily reflect the views nor behaviours of patients/women. ” just below the heading “**Results of the Stakeholder dissemination workshop discussions”**
iii) We also added the words “providers perceived” in several sentences relating to provider perceptions of barriers within the results section
iv) We emphasized that results reflected provider perceptions in the amended Figure 2
v) We added lines 656-660: “These determinants need verification and further exploration among multiple stakeholder groups to understand how best to increase provision (supply) and demand for cancer screening services. Our findings highlight the need for further engagement and research among community, patient and provider stakeholder groups to further understand determinants and opportunities to increase screening rates.”
